# *Bacillus Calmette–Guérin* Vaccination Promotes Efficient and Comprehensive Immune Modulation in Guinea Pig Models

**DOI:** 10.3390/vaccines13030305

**Published:** 2025-03-12

**Authors:** In-Ohk Ouh, Min Jung Kim, Kwangwook Kim, Heeji Lim, Ye Jin Yang, Ji Woong Heo, Han Nim Choi, Hun Hwan Kim, Hu-Jang Lee, Phil-Ok Koh, Seo Young Moon, Eun Bee Choi, Yoo-Kyung Lee, Kwang Il Park

**Affiliations:** 1 Division of Vaccine Development Coordination, Center for Vaccine Research National Institute of Infectious Diseses, Korea National Institute of Health, Korea Disease Control and Prevention Agency, Cheongju-si 28159, Republic of Korea; dvmoio@korea.kr (I.-O.O.); kwkim83@korea.kr (K.K.); dalgi0519@korea.kr (H.L.); msy1477@korea.kr (S.Y.M.); dmsql2274@korea.kr (E.B.C.); 2 College of Veterinary Medicine, Gyeongsang National University, Gazwa, Jinju 52828, Republic of Korea; minjung0102@gnu.ac.kr (M.J.K.); yang93810@gnu.ac.kr (Y.J.Y.); hujiw7806@gnu.ac.kr (J.W.H.); vethan99@gnu.ac.kr (H.N.C.); hujang@gnu.ac.kr (H.-J.L.); pokoh@gnu.ac.kr (P.-O.K.); 3 Department of Physiology and Aging, College of Medicine, Institute on Aging, University of Florida, Gainesville, FL 32610, USA; kim.hunhwan@ufl.edu

**Keywords:** *Mycobacterium tuberculosis*, *Bacillus Calmette–Guérin*, hematoxylin and eosin, immunohistochemistry, guinea pigs

## Abstract

**Background/Objectives:** *Tuberculosis* (TB), caused by *Mycobacterium tuberculosis H37Rv (M. tuberculosis*), primarily affects the lungs. The *Bacillus Calmette–Guérin* (BCG) vaccine is the only available TB vaccine. Guinea pigs serve as an excellent preclinical model due to the similarity to human *Tuberculosis* pathology. However, the lack of a standardized vaccination protocol in guinea pigs causes inconsistencies in efficacy assessments, limiting precise evaluation and its application in vaccine studies. This study aims to address this gap by establishing a consistent and reliable protocol for evaluating the immunological efficacy of BCG vaccination. **Methods:** Guinea pigs were divided into control, *M. tuberculosis*-infected, and BCG-vaccinated groups. Four weeks post-vaccination, the infected and vaccinated groups were challenged with *M. tuberculosis*. The bacterial burden in the lungs and spleen was measured, histopathological changes were analyzed using hematoxylin and eosin (H&E) staining, and the infection levels of *M. tuberculosis*, as well as the presence of interleukin-2 (IL-2), tumor necrosis factor-alpha (TNF-α), and interferon-gamma (IFN-γ) positive cells, were evaluated through immunohistochemical (IHC) staining. **Results:** BCG vaccination reduced the bacterial load to 3.60 × 10^4^ CFU/lung and 5.52 × 10^3^ CFU/spleen compared to 3.78 × 10^5^ CFU/lung and 1.54 × 10^4^ CFU/spleen in the infected group. The mean histopathological score for lungs was 1.67 compared to 2.67 in the infected group. Similarly, the mean histopathological score for the spleen was 1.33 compared to 2.33 in the infected group. IHC analysis showed a notable reduction in *M. tuberculosis* and inflammatory cytokine-positive cells in the vaccinated group. The TNF-α, IL-2, and IFN-γ staining intensity decreased by 9.3, 4.8, and 11, respectively, compared to the infected group. **Conclusions**: This protocol enhances consistency in vaccine assessments, providing a reliable benchmark for the development of safer, more effective, and accessible TB vaccines.

## 1. Introduction

*Tuberculosis* (TB) remains a major global public health challenge, requiring continuous efforts for effective prevention and control [1]. In recent decades, there has been a global decrease in the prevalence of TB infection, though it remains a significant public health issue in numerous nations [2]. This disease is a respiratory infection that is spread through airborne transmission, with infection and prevalence rates being directly linked to the transmission of the *tuberculosis* bacillus. Despite efforts to control the disease, TB remains prevalent in numerous nations, particularly in regions with high transmission rates. In recent decades, there has been a global decrease in the prevalence of *tuberculosis* infection; however, challenges such as drug-resistant strains and inconsistent vaccine efficacy continue to hinder complete eradication [3]. The *Bacillus Calmette–Guérin* (BCG) vaccine is employed for TB prevention and is produced from a weakened strain of *Mycobacterium bovis* [4]. This vaccine elicits a targeted T cell response against *Mycobacterium tuberculosis H37Rv* (*M. tuberculosis*), and it is utilized to prevent or reduce the occurrence of TB infection. While BCG vaccination provides protection against severe forms of TB in children, its efficacy in preventing pulmonary TB in adults varies significantly depending on geographical region and individual immune response [5]. Consequently, further research is needed to evaluate its immunological effects and potential for improving *TB* control strategies. Furthermore, *H37Rv* is a commonly utilized research strain of *M. tuberculosis* in studies related to TB [6]. *M. tuberculosis* is utilized as a pivotal model for examining the pathological features and immune responses linked to TB [7]. T cells respond to infection via the TB bacillus by secreting various cytokines, such as Interferon-γ (IFN-γ), which enhance the immune response against the bacillus [8]. Specifically, CD4^+^ T cells become activated through the recognition of TB antigens displayed by antigen-presenting cells, whereas CD8^+^ T cells are involved in the identification and removal of infected cells [9,10]. The BCG vaccine is used globally and is predominantly given to infants and young children [11]. The timing of the BCG vaccination is determined based on local TB epidemiology, vaccination program guidelines, and the child’s health status [4].

In regions with a high prevalence of TB, it is advisable to provide the BCG vaccine during the early postnatal period. Studies on TB immunology primarily involve infecting small animals such as mice, rats, guinea pigs, and rabbits. The lung structure and immune system of guinea pigs closely resemble those of humans, making them a valuable model in the study of pulmonary infections caused by various strains of TB [12,13]. Research utilizing guinea pigs contributes to understanding the pathological and immunological aspects of TB, as the primary infection site is in the lungs [13].

The aim of our research is to study T cell responses in the lungs and spleen in order to evaluate how effective the BCG vaccine is when administered to guinea pigs four weeks before infection with TB. By considering variations in BCG efficacy and its inconsistent global usage, our study can provide insights into its immunological mechanisms and explore its potential role in improving TB control strategies worldwide. This research contributes to the modeling of human immune system responses and assists in the development of vaccines.

## 2. Materials and Methods

### 2.1. Animals

Female 7-week-old Hartley guinea pigs were obtained from Samtaco (Osan, Republic of Korea). The animals were kept at a temperature of 22  ±  2 °C and a relative humidity of 50  ±  5% in a 12 h light/dark cycle, being fed guinea pig chow and water ad libitum. This animal experiment complied with the guidelines of the Institutional Animal Care and Use Committee of Gyeongsang National University (Jinju, Republic of Korea, GNU-220920-E0114).

### 2.2. Bacterial Culture

*M. tuberculosis* and *Mycobacterium bovis* BCG Pasteur 1173P2 were cultured in sealed conditions on a Middlebrook 7H10 agar medium at 37 °C for 2–3 weeks. Colonies were then transferred to 10 mL of the Middlebrook 7H9 broth medium and cultured for 1 week. The seed culture was inoculated into 400 mL of Middlebrook 7H9 broth in a 1 L Erlenmeyer flask supplemented with 10% OADC and 0.05% Tween 80 and cultured at 37 °C for another week until the optical density (OD600) reached 0.6–0.8 in the mid-log growth stage. The harvested bacteria tend to form clumps and so were passed through a 10 μm cell strainer to isolate single bacterial cells. The resulting suspension was combined with 8 mL of Middlebrook 7H9 broth (10% OADC + 0.05% Tween 80) and 2 mL of 20% glycerol, thoroughly mixed, and aliquoted into 1 mL volumes in sterile stock tubes. The aliquots were stored at −70 °C for long-term preservation.

### 2.3. Bacterial Strains and Vaccines

Guinea pigs were acclimatized to laboratory conditions for one week before the experiment. Twenty-four guinea pigs were then randomly divided into three experimental groups. The control group (4 guinea pigs) received 200 µL of PBS, a BCG group (10 guinea pigs) was vaccinated subcutaneously with 200 μL of BCG at 5 × 10^4^ CFU, and an infection-only group (10 guinea pigs) was infected with *M. tuberculosis* H37Rv without BCG vaccination.

Four weeks after the BCG (*Mycobacterium bovis* BCG Pasteur 1173P_2_) vaccination, both the BCG and infection-only groups were infected with *M. tuberculosis* H37Rv via aerosol exposure. To assess the infection progression and immune response, five guinea pigs from each infected group were sacrificed one day after 4 weeks of infection to measure the bacterial load (CFU) in the lungs. The remaining animals were sacrificed four weeks post-infection to reassess the CFU counts and perform histopathological analysis.

### 2.4. Infection and Survival Studies

Four weeks after immunization, the BCG-vaccinated and infection-only groups were exposed to *M. tuberculosis* through the respiratory route using a Glas-Col^®^ aerosol exposure chamber system (Glas-Col, Terre Haute, IN, USA). The bacterial suspension was prepared by diluting working stocks to 3 × 10^2^ CFU/mL in sterile distilled water and placed in the nebulizer jar. The guinea pigs were exposed to the aerosol for 30 min. The control group and age-matched guinea pigs that were 4 weeks old at the time of BCG immunization were also infected with *M. tuberculosis* via aerosol exposure.

Four weeks post-infection, all remaining guinea pigs were sacrificed. The lungs and spleens were collected, homogenized, and serially diluted (10^2^–10^4^ CFU/mL). The samples were plated on the 7H10 agar medium to assess the bacterial burden and evaluate the ability to inhibit *M. tuberculosis* growth. Histopathological analysis was also performed to further assess the infection severity (Table 1).

### 2.5. Hematoxylin and Eosin (H&E) Staining

Tissue sections that were 4 μm thick were prepared and stained using H&E. The lung and spleen were examined for histopathological changes using a Nikon ECLIPSE Ni-U light microscope (Nikon, Tokyo, Japan). The thickness of the alveolar epithelium was measured in ten randomly chosen areas per guinea pig (×100) using Image J software (v46a; NIH, Bethesda, MD, USA). The grading of granulomatous lesions was determined by quantifying the number of inflammatory cells and analyzing the distribution pattern inside the liver and spleen. For the grading of granulomatous lesions, three random fields were selected at ×100 magnification, and the number of granulomatous lesions was counted. Concisely, we evaluated the pathology of the lung and spleen sections to determine a combined score (Table 2). Histopathological evaluations were carried out in a ISO9001:2015 [14] and GLP compliant laboratory and evaluated by two veterinary pathologists blinded to the animal details and methodology.

### 2.6. Immunohistochemical (IHC) Staining

The paraffin-embedded slides were de-paraffinized and rehydrated. Antigen retrieval was performed using a citrate buffer in a high-pressure cooking pot, followed by the quenching of endogenous peroxidase and blocking with normal serum. The slides were incubated overnight at 4 °C with primary antibodies, anti-TNF-α, anti-IL-2, anti-IFN-γ (1:100, Invitrogen, Waltham, MA, USA), and anti-*M. tuberculosis* (1:100, Abcam, Cambridge, UK). Following this, the slides were washed in PBS and incubated for 2 h at room temperature with either a rabbit anti-rat IgG or goat anti-rabbit IgG biotinylated secondary antibody (1:100, Vector Laboratories, Burlingame, CA, USA), followed by another PBS wash. The slides were then treated with an AB complex and developed into a chromogen with diaminobenzidine (DAB; Vector Laboratories, CA, USA). Cells with brown nuclei were identified, and the number of controls was counted and expressed as a percentage of total cells.

IHC evaluations were performed in an ISO9001:2015 and GLP-compliant laboratory and evaluated by two veterinary pathologists blinded to animal details and methodology. Additionally, for IHC evaluation, the positive cells for each antibody were counted, and the average value was displayed in at least six randomly selected areas from three tissue slides.

### 2.7. Statistical Analysis

Values are expressed as means ± standard error of the mean (SEM). Multiple comparisons were performed using one-way ANOVA, followed by the Tukey–Kramer post hoc test. All statistical analyses were performed using GraphPad Prism ver. 8.02 software (GraphPad Software, Inc., San Diego, CA, USA), and a *p* < 0.05 was considered statistically significant.

## 3. Results

### 3.1. Vaccination Results in a Confirmed Inhibition of M. tuberculosis Growth

Both guinea pigs from the control group and those that were 4 weeks old during the BCG immunization period were exposed to *M. tuberculosis* via air infection, and the ability to inhibit the growth of *M. tuberculosis* was then confirmed.

**Figure 1:**
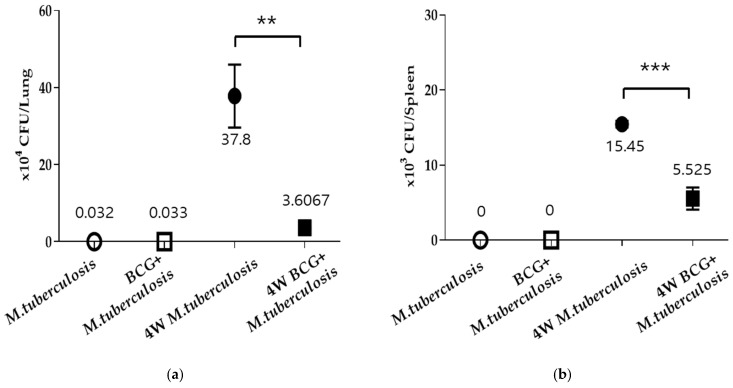
BCG vaccination inhibits the growth of *M. tuberculosis* in the lungs and spleen of guinea pigs. After inducing airborne infection with tuberculous bacilli in guinea pigs from both the RV group and those immunized with BCG for four weeks, the animals were sacrificed. *M. tuberculosis* growth was measured as BCG-naive or BCG-vaccinated in the (**a**) lung and (**b**) spleen. Data represent means ± SEM (ANOVA, ** *p* < 0.01, *** *p* < 0.001).

A notable bacterial load enhancement in the growth of TB bacteria in the lungs and spleens was observed in the *M. tuberculosis* group of guinea pigs, whereas a clear bacterial load reduction in TB bacteria growth was evident in the guinea pigs immunized with BCG for four weeks (Figure 1). Thus, although this research did not verify variances in the suppression of TB bacteria growth according to the length of BCG immunization, it did demonstrate that the immunological effectiveness of BCG played a role in the inhibitory impact on bacterial growth.

### 3.2. Histopathological Changes Following BCG Vaccination in Lung and Spleen of Guinea Pig

**Figure 2:**
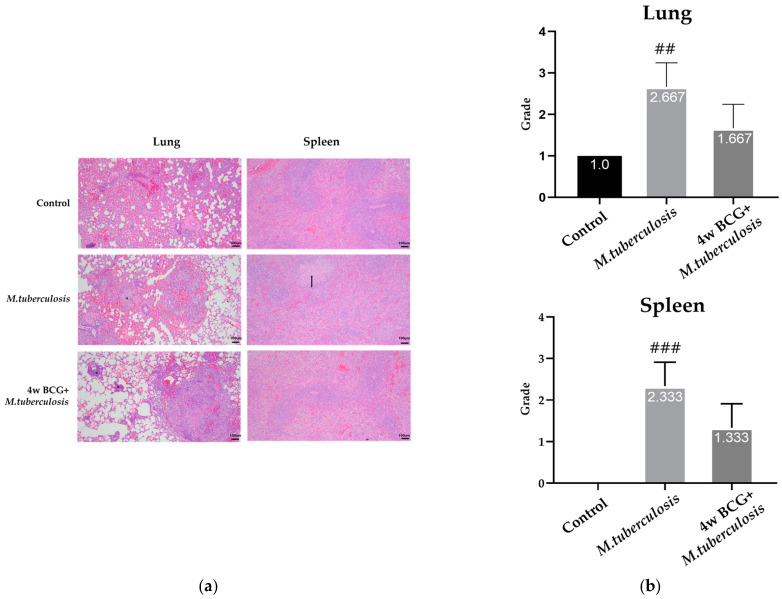
Changes in lung and spleen pathology in *M. tuberculosis* infection and BCG-vaccinated guinea pigs. Representative photomicrographs from sections of formalin-fixed and paraffin-embedded lung and spleen tissues collected 4 weeks after the *M. tuberculosis* challenge are shown. (**a**) Morphological assessments were performed using H&E staining in lungs and spleen tissues. (**b**) Quantitative analysis of inflammation grading in lung and spleen shows mean ± SEM scores for each group, analyzed using ANOVA (^##^ *p* < 0.01, ^###^ *p* < 0.001). Scale bar = 100 µm. *: marginal zone cellularity increases, ↑: macrophage aggregation increases.

The histological results from the H&E staining identified the control group, and granulomatous inflammation was observed, as characterized by organized clusters of immune cells. In the RV group, acute broncho-alveolar inflammation was prominent and primarily composed of macrophages. This inflammation was diffused and accompanied by notable congestion in perivascular regions and alveolar spaces, without any clear compartmentalization. Edema was also present, which is indicative of an acute inflammatory response to infection. In the BCG + RV group, similar histological features to those observed in the control group were noted, without any significant differences in granulomatous formation or the distribution of inflammatory cells. In the control group, the histological structure of the spleen remained consistent with normal spleen architecture, showing no significant abnormalities. In the RV group, the sporadic aggregation of immune cells was observed in both the white and red pulp, suggesting the localized activation of immune responses. In the BCG + RV group, similar structural features to the control group were seen, though there was a mild increase in lymphocyte apoptosis (Figure 2).

The histological images of the lung and spleen indicate that combining BCG with RV leads to a decrease in inflammation and granuloma formation in comparison to RV alone. This suggests that BCG may have a regulatory impact on the inflammatory response through its immunomodulatory properties, resulting in a more regulated immune reaction in both organs.

### 3.3. Analysis of the Infection Level of M. tuberculosis Using Immunohistochemical Staining in Guinea Pig Lung and Spleen

**Figure 3:**
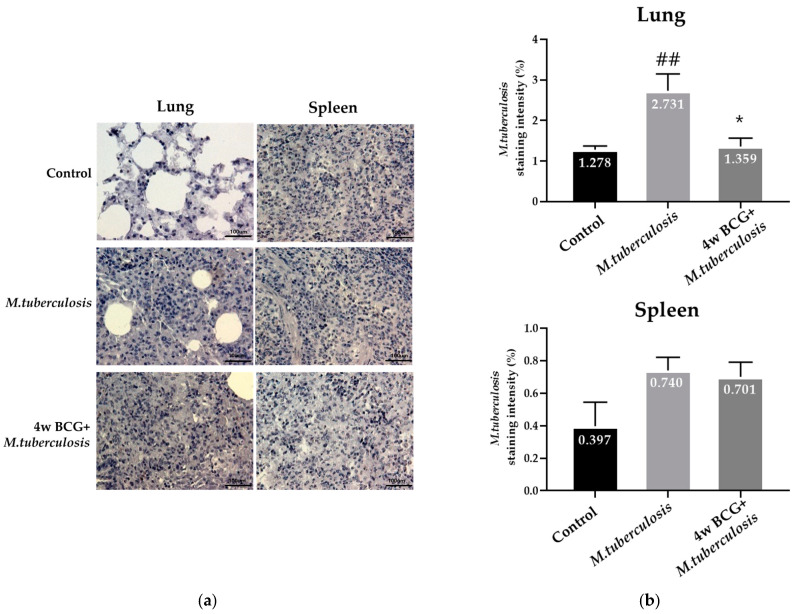
IHC staining of cell populations in the lung and spleen level of *M. tuberculosis*. (**a**) Representative photomicrographs from lung sections after 4 weeks of *M. tuberculosis* infection in the lung (left panels) and the spleen (right panels) groups showing staining patterns for IHC analysis of the tissue (**b**) % *M. tuberculosis*-positive cells (*Y*-axis) over the control, and groups on the *X*-axis. Data represent mean ± SEM (ANOVA, ^##^ *p* < 0.01, * *p* < 0.05). Scale bar = 100 μm.

The IHC staining of anti-*M. tuberculosis* antibodies was used to confirm and analyze the extent of infection by the TB bacteria. IHC staining revealed an increase in the number of *M. tuberculosis* in the lung tissue of the RV group of guinea pigs. In contrast, a decrease in the number of *M. tuberculosis* was observed in the group that received the BCG vaccine and were exposed to *M. tuberculosis* four weeks later. No notable differences were observed in the spleen tissue between the groups (Figure 3).

### 3.4. IHC Analysis of TNF-α, IL-2 and IFN-γ Positive Cells in Guinea Pig Lung

**Figure 4:**
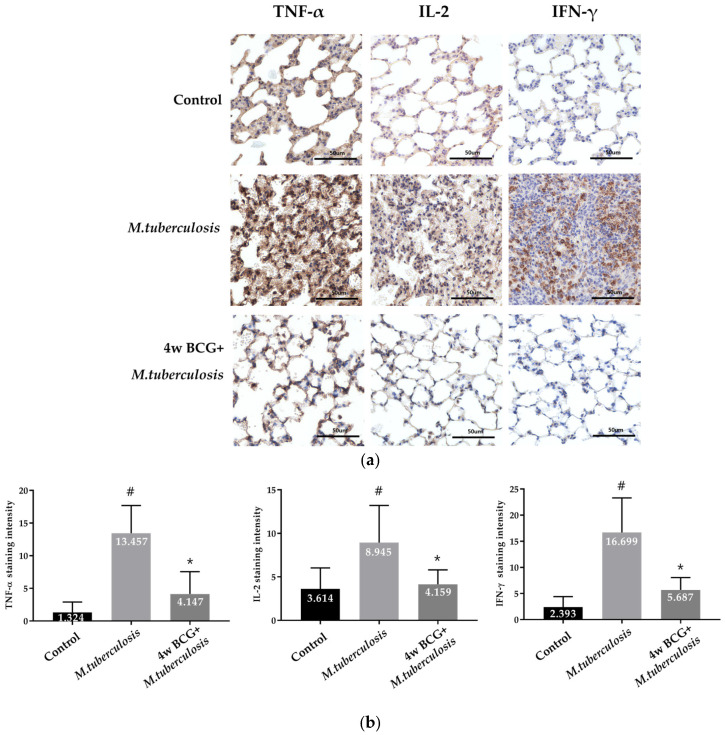
IHC staining of cell populations in the lungs of guinea pigs. Representative photomicrographs of lung sections after 4 weeks of *M. tuberculosis* infection show staining patterns for IHC analysis of the lung. (**a**) Confirmation and analysis of lymphocyte distribution through tissue IHC of T lymphocyte markers (TNF-α, IL-2, and IFN-γ). (**b**) T lymphocyte markers Staining intensity (*Y*-axis) over the control, and groups on the *X*-axis. Data represent mean ± SEM (ANOVA, ^#^ *p* < 0.05, * *p* < 0.05). Scale bar = 50 μm.

The distribution of lymphocytes in guinea pigs was confirmed through the IHC staining of tissues targeting TNF-α, IL-2, and IFN-γ. In the RV group, there was a notable increase in TNF-α, IL-2, and IFN-γ expression, reflecting an acute inflammatory response. However, in the group pre-vaccinated with BCG before *M. tuberculosis* infection, a statistically significant reduction in TNF-α expression was observed, along with a marked decrease in IL-2 and IFN-γ levels. These findings suggest that BCG vaccination effectively modulates the immune response, significantly reducing the expression of key pro-inflammatory cytokines following *M. tuberculosis* exposure (Figure 4).

## 4. Discussion

When an individual is infected with *M. tuberculosis*, the immune response initiates in the lungs, with alveolar epithelial cells acting as the primary defense mechanism [15,16]. After being inhaled, the bacteria enter the alveoli and come into contact with alveolar macrophages, which phagocytose the bacteria and trigger an inflammatory reaction, thus attracting components of the innate immune system, such as natural killer cells, neutrophils, and innate lymphoid cells, to combat the pathogen [17]. When initial immune responses fail to eradicate the bacteria, the adaptive immune system is activated, involving T cells and B cells in order to enhance cellular and humoral immunity against *M. tuberculosis* [18,19,20].

While the immune response is strong, the majority of individuals (90–95%) infected will develop latent tuberculosis infection (LTBI), in which the pathogen remains dormant without causing active disease [21]. This delay highlights the significance of efficacious vaccines.

The *Bacillus Calmette–Guérin* (BCG) vaccine is currently the sole authorized vaccine for *tuberculosis* and is extensively utilized because of its protective potency and minimal adverse reactions [22]. BCG vaccination plays a crucial role in both preventing and treating *tuberculosis*, particularly by decreasing bacterial levels during infection and improving immune responses through cytokine modulation and cellular recruitment. Recent research highlights the effectiveness of BCG in controlling *M. tuberculosis* growth, particularly in animal models [23,24]. In guinea pigs, BCG vaccination followed by exposure to *M. tuberculosis* led to significant suppression of bacterial growth in the lungs and spleen, demonstrating its efficacy not only in preventing active infection but also in reducing the risk of reactivation (Figure 1). The immunological advantages of BCG establish its significance in the management of TB on a global scale, showcasing its effectiveness in both controlling active infection and lowering the risk of reactivation in LTBI.

H&E staining is a foundational method in pathology for evaluating structural and inflammatory changes in tissues, as well as for presenting visual cues of disease advancement, treatment effectiveness, and immune modulation in diverse circumstances [25]. Normal tissue-like findings were observed in the well-structured lung and spleen tissue of guinea pigs in the control group, with no significant inflammation noted. The group infected with RV exhibited significant inflammation in the broncho-alveolar region, characterized by the widespread infiltration of macrophages and the pronounced congestion of the perivascular and alveolar spaces with edema, which is indicative of an acute inflammatory reaction [26,27]. On the other hand, the BCG + RV group exhibited histological characteristics resembling those of the control group, including organized granulomas and a lack of substantial changes in inflammatory cell dispersion or tissue morphology, albeit with mild lymphocyte apoptosis in the spleen. This suggests a well-balanced immune modulation rather than simple suppression, thus ensuring a controlled inflammatory response without impairing protective immunity. In addition, this indicates that the inflammatory response may be regulated without risking heightened immune activation (Figure 2). These findings suggest that BCG vaccination not only preserves tissue integrity but also modulates immune activation, preventing excessive inflammation while maintaining pathogen control.

The lungs are the main organ targeted by *M. tuberculosis*, and promoting localized *M. tuberculosis*-specific immunity in the lungs is essential for managing *M. tuberculosis* MTB infection [28,29,30]. IHC analysis revealed a greater concentration of *M. tuberculosis* antigens in the lung tissues of the RV group as opposed to the BCG + RV group, as illustrated in Figure 3. This discrepancy indicates that BCG vaccination decreases the bacterial burden in lung tissue, aligning with prior research that has indicated a lower prevalence of bacterial colonies in primary *M. tuberculosis* MTB infection sites, such as the lungs, in animals vaccinated with BCG. While no significant difference in the bacterial load was observed in the spleen, this may suggest that BCG’s protective effect is localized to primary infection sites, such as the lungs, rather than systemic *M. tuberculosis* reduction.

When the inherent immune response is ineffective in eradicating the invading *M. tuberculosis* MTB, CD8^+^ T cells and Th1-type cell-mediated immune responses are initiated [31,32]. These responses secrete cytokines, particularly IFN-γ, which in turn stimulate the macrophage system to eliminate the bacteria residing within the macrophages and forming granulomas to regulate *M. tuberculosis* infection MTB [33]. IHC analysis revealed a significant increase in the expression of the cytokines TNF-α, IL-2, and IFN-γ in lung tissues of the RV group, suggesting a robust inflammatory response to the infection. In contrast, the BCG + RV group exhibited a notable decrease in the expression levels of TNF-α, IL-2, and IFN-γ, as shown in Figure 4. A well-regulated cytokine response is critical for balancing pathogen clearance and preventing immunopathology. Overproduction may cause tissue damage, while insufficient levels can allow bacterial persistence. Future research should investigate optimized BCG vaccination strategies and novel *TB* vaccines to enhance long-term immunity and protective efficacy. These results are consistent with reports [32,34,35] indicating that BCG influences the immune response in order to prevent excessive inflammation and confer protective immunity against *M. tuberculosis*.

This study offers an analysis on the protective benefits of BCG vaccination against *M. tuberculosis* infection in guinea pigs, with a specific focus on bacterial growth inhibition, histopathological changes, infection levels in tissues, and cytokine expression as indicators of immune modulation. Moreover, this study illustrates that the BCG challenge-based model is a practical and effective approach for evaluating vaccine candidates’ effectiveness. Subsequent studies, particularly considering the advancement of novel TB vaccines designed to improve or substitute BCG, could explore the effects of various BCG vaccination procedures on enduring immunity and safeguarding.

## 5. Conclusions

Our research has showed that a robust immune reaction to *M. tuberculosis* manifests within a 4-week period post-BCG vaccination in a guinea pig experimental model. In order to enhance the effectiveness and cost-efficiency of vaccine development, we suggest the establishment of a standardized BCG efficacy index that has the potential to reduce the duration of preliminary animal testing. Enhancing the accuracy and safety of BCG-related immunogenicity assessments could facilitate the systematic evaluation of next-generation vaccine candidates, ultimately accelerating the development of more efficient, cost-effective, and safer TB vaccination strategies.

## Figures and Tables

**Table 1:** Airborne *M. tuberculosis* infection procedure.

Steps	Function	Time
1	Pre-heating	15 min
2	Nebulizer	30 min
3	Cloud decay	30 min
4	UV	15 min
5	Cool down	10 min
Total Times		1 h 40 min

**Table 2:** Evaluate inflammation in the lungs and spleen of a guinea pig.

Grade	
0	Normal(No changes in the cells or tissue structure. No increased apoptosis or macrophage aggregation)
1	Minimal (Slight cell increase, but barely noticeable. Rare apoptotic cells and small macrophage clusters, with no impact on structure)
2	Slight (Mild cell increase, structure intact. Mild apoptosis, occasional cell clusters, and mild macrophage aggregation)
3	Moderate(Increase in cellularity at the margins, and a rise in macrophage aggregation)
4	Severe (Large cell clusters, showing significant tissue damage. Extensive apoptosis, lymphocyte loss, and marked cellular infiltration and structural disruption)

## Data Availability

The original contributions presented in this study are included in the article. Further inquiries can be directed to the corresponding author(s).
